# PMAxx dye-assisted colorimetric dual DNAzyme-LAMP (PD-cDDLAMP) for rapid phenotypic antimicrobial susceptibility testing of *Escherichia coli*

**DOI:** 10.1128/spectrum.01212-26

**Published:** 2026-09-02

**Authors:** Alaa H. Sewid, Joseph H. Ramos, Benti D. Gelalcha, Yuri Matsuoka, Oudessa Kerro Dego, Shigetoshi Eda

**Affiliations:** 1 School of Natural Resources, The University of Tennessee Institute of Agriculture 4292, Knoxville, Tennessee, USA; 2 Department of Microbiology, Faculty of Veterinary Medicine, Zagazig University 68799, Zagazig, Egypt; 3 Department of Biomedical and Diagnostic Sciences, College of Veterinary Medicine, The University of Tennessee Institute of Agriculture 4292, Knoxville, Tennessee, USA; 4 Departments of Animal Science, The University of Tennessee Institute of Agriculture 4292, Knoxville, Tennessee, USA; University of Georgia College of Veterinary Medicine, Athens, Georgia, USA

**Keywords:** antimicrobial susceptibility testing, antimicrobial resistance, PMAxx, DNAzyme-LAMP

## Abstract

Rapid antimicrobial susceptibility testing (AST) is crucial for combating antimicrobial resistance and guiding effective therapy. Conventional phenotypic AST methods are often time-consuming, requiring 18–72 h for results, while genotypic approaches often depend on pre-existing knowledge of resistance markers. Although nucleic acid-based phenotypic AST methods have reduced turnaround time, attempts to shorten antibiotic exposure often produce only small differences between susceptible and resistant bacteria. Consequently, most loop-mediated isothermal amplification (LAMP)-based phenotypic AST assays still require prolonged antibiotic exposure (up to 4 h) or rely on sophisticated high-resolution nucleic acid detection, such as digital LAMP, to accurately detect these subtle changes, limiting their simplicity. Therefore, there is a critical need to develop a rapid assay with both short antibiotic exposure times and a simple readout. To address this unmet need, we developed PMAxx dye-assisted colorimetric Dual DNAzyme-LAMP (PD-cDDLAMP), a rapid nucleic acid-based phenotypic AST method that enhances suppression of nucleic acid amplification from antibiotic-susceptible bacteria through the selective binding of photoreactive DNA-crosslinker dye (PMAxx) to the DNA of membrane-compromised cells. This strategy enables reliable discrimination between antibiotic-resistant bacteria (“signal-on”) and antibiotic-susceptible bacteria (“signal-off”) after only 30 min of antibiotic exposure using a user-friendly colorimetric DNAzyme-LAMP readout system. PD-cDDLAMP achieved 91.7% sensitivity, 83.3% specificity, and 87.5% accuracy in detecting *Escherichia coli* exposed to ampicillin and tetracycline, outperforming current methods by reducing antibiotic exposure time to just 30 min. The method was further validated in spiked milk samples, demonstrating its potential for on-site AST in dairy farming. PD-cDDLAMP’s simplicity, speed, and reduced workflow complexity make it a promising tool for rapid AST.

**IMPORTANCE:**

Rapid, reliable antimicrobial susceptibility testing (AST) is essential for guiding targeted therapy and combating the escalating threat of antimicrobial resistance (AMR). However, current phenotypic ASTs are too slow for urgent decision-making, and genotypic assays cannot fully capture emerging or unknown resistance mechanisms. The PMAxx dye-assisted colorimetric dual DNAzyme-loop-mediated isothermal amplification (PD-cDDLAMP) assay introduced in this work provides a rapid nucleic acid-based phenotypic AST capable of distinguishing antibiotic-resistant from susceptible bacteria within 30 min of antibiotic exposure. By combining PMAxx-mediated suppression of nucleic acid amplification in susceptible cells with equipment-free colorimetric DNAzyme-LAMP readout, PD-cDDLAMP bridges the gap between speed and phenotypic accuracy. Its high diagnostic performance, compatibility with complex matrices such as milk, and minimal instrumentation requirements position this method as a promising point-of-care tool for clinical microbiology laboratories and on-site testing environments. PD-cDDLAMP has the potential to significantly shorten time-to-result while maintaining reliability, thereby supporting antimicrobial stewardship.

## INTRODUCTION

The emergence and spread of antimicrobial resistance (AMR) poses a significant public health challenge, contributing to millions of annual deaths worldwide (1). Accurate and rapid antimicrobial susceptibility testing (AST) is critical for guiding effective treatment, reducing the reliance on broad-spectrum antibiotics, and combating AMR (2). Conventional phenotypic ASTs, which rely on detecting bacterial growth in the presence or absence of antibiotics, typically require lengthy incubation periods of 18–72 h for reliable results (3). This delays critical treatment decisions.

While genotypic ASTs offer accelerated results (within hours) (4) by identifying specific resistance genes or mutations (5), they are limited by their inability to identify novel resistance mechanisms and their reliance on pre-existing knowledge of resistance markers, which may not always correlate with phenotypic resistance (6). Moreover, these tests have not been implemented more broadly due to their lack of generalizability across different pathogens and mechanisms of resistance, particularly in the case of Gram-negative bacteria, where more than 800 resistance genes are known for β-lactam class antibiotics alone with the continual emergence of new resistance genes (7), which complicates the design of a comprehensive genotypic AST, even for a single organism.

Phenotypic AST remains the most reliable approach, as it directly assesses bacterial response to antibiotics (8), but its slow turnaround time hinders its utility in urgent clinical settings (2). Therefore, there is an imperative need for innovative diagnostic AST that can combine the rapidity of genotypic methods with the reliability of phenotypic assays (9). Nucleic acid (NA)-based phenotypic AST techniques leverage molecular techniques to assess AMR by detecting changes in bacterial DNA replication, or amplification in response to antibiotics (10, 11). This approach has enabled the development of rapid and precise ASTs that can facilitate timely decisions in antibiotic treatment and improve patient outcomes in different contexts (9, 11–18).

Despite these advances, current NA-based phenotypic AST methods still face key limitations that hinder their translation into practical and rapid testing platforms (8). For instance, loop-mediated isothermal amplification (LAMP)-based phenotypic AST methods require prolonged antibiotic exposure time (up to 4 h) to evaluate the threshold time difference (ΔTt) in DNA amplification between antibiotic-free and antibiotic-exposed samples (14). However, reducing the antibiotic exposure time typically results in smaller differences in DNA abundance between antibiotic-treated and control samples, which are difficult to resolve using conventional readout methods (11). As a result, high-resolution NA detection methods, such as quantitative PCR (qPCR), digital PCR, or digital LAMP, are required to accurately quantify these subtle changes within a reduced exposure time (13, 19, 20). While effective, these methods introduce additional complexity, require specialized instrumentation, and reduce workflow simplicity, thereby limiting their broader applicability.

In addition, several reported AST strategies rely on multi-step workflows and complex signal acquisition systems. These include assessments of DNA or polymerase accessibility with or without surfactant-mediated cell wall permeabilization (15, 16), barcode-based sequencing approaches requiring labeling, amplification, and pyrosequencing readout (12), and cationic dye-triggered electrokinetic gold nanoparticle agglutination (CD-TEAA), which relies on DNA-functionalized gold nanoparticles, cationic dyes, and microfluidic electrokinetic stacking for 16S rRNA detection (9). Collectively, these approaches further increase procedural and analytical complexity.

Together, these limitations highlight two major unresolved challenges in current NA-based phenotypic AST: (i) the need to reduce antibiotic exposure time while maintaining sufficient signal discrimination, and (ii) the need for simplified workflows with user-friendly readout systems.

The photoreactive DNA-crosslinker dye, PMA or PMAxx (improved propidium monoazide), has the ability to selectively bind to the DNA of dead or membrane-compromised cells (15), inhibiting PCR amplification (21). These dyes have been applied in microbial viability studies (22) and in rapid NA-based phenotypic AST, including studies in *Mycobacterium tuberculosis* and *Neisseria gonorrhoeae* (17, 23), as well as in nanopore-based 16S rRNA metagenome profiling of drug-resistant bacteria (18).

However, challenges related to workflow complexity, instrumentation requirements, and readout simplicity remain insufficiently addressed. Therefore, there is an urgent need for rapid isothermal amplification-based AST platforms that integrate strong suppression of nonviable cell signals with simple, user-friendly detection systems (11).

In 2022, Song et al. reported a LAMP combined with a DNA-based peroxidase-mimicking enzyme (DNAzyme), termed as DNAzyme-LAMP, which enabled a colorimetric detection method for SARS-CoV-2 viruses (24). We have also previously modified, optimized, and validated a DNAzyme-LAMP assay for the detection of *Escherichia coli* in vegetable and milk samples. As the new DNAzyme-LAMP produces amplicons that contain two types of DNAzymes to produce a colorimetric signal, we named it colorimetric dual DNAzyme LAMP (cDDLAMP). The limit of detection (LOD) of the cDDLAMP was 6.3 colony-forming units (CFU) or 0.1 aM gene copies (25). In a following study, we also demonstrated that cDDLAMP was able to detect three genes (*stx1*, *stx2*, and *eae*) of Shiga toxin-producing *E. coli* in milk and vegetable rinse water with a LOD of 1 CFU/mL (26).

**Fig 1:**
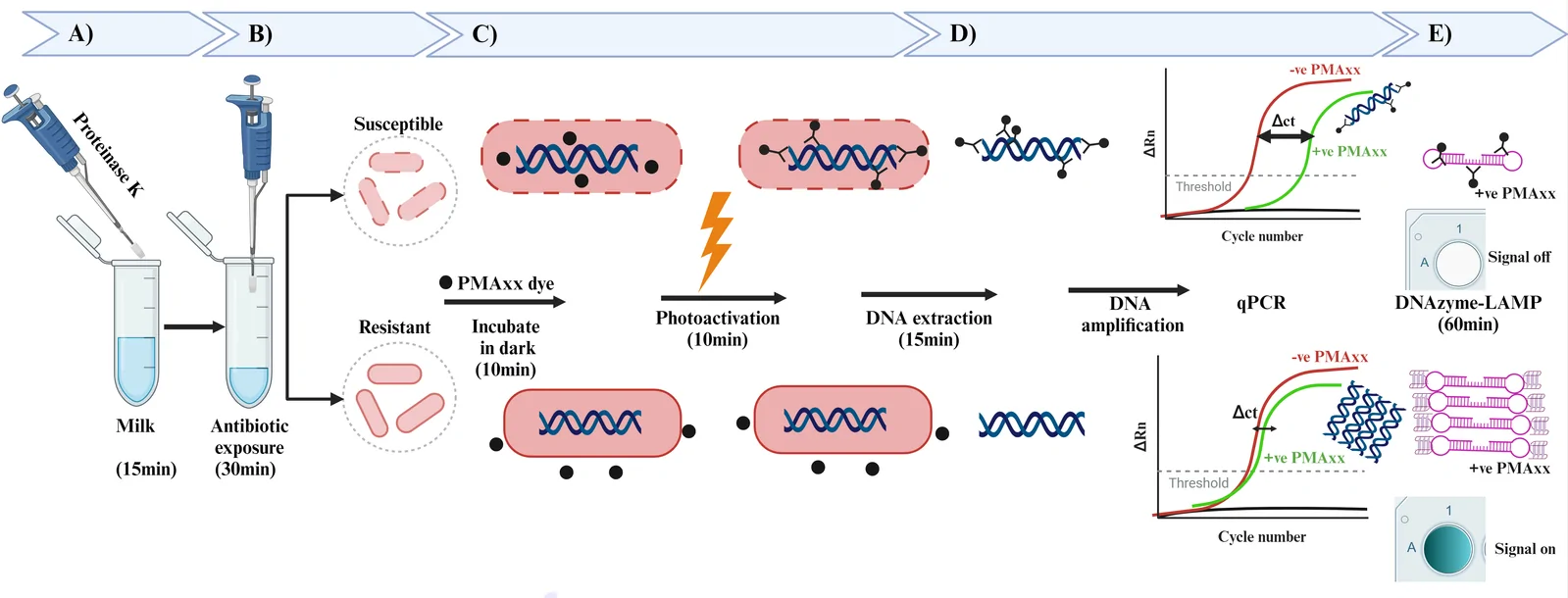
Proposed clinical workflow for PD-cDDLAMP in antimicrobial susceptibility testing based on phenotypic changes in bacteria following antibiotic treatment. In the proposed workflow, milk samples are collected from cows with suspected *E. coli* infections. (**A**) Initial processing involves proteinase K treatment for 15 min to digest milk proteins, allowing purification of *E. coli*. (**B**) The resulting *E. coli* pellet is then incubated with the selected antibiotic for 30 min to assess susceptibility. (**C**) Cells are treated with PMAxx for 10 min in the dark, followed by a 10-min photoactivation step to enable PMAxx to selectively bind to DNA from membrane-compromised cells. (**D**) Genomic DNA is extracted by heat lysis (15 min), and quantitative PCR (qPCR) is performed. In resistant strains, PMAxx treatment yields similar Ct values to untreated controls, whereas in susceptible strains, PMAxx treatment results in significantly higher Ct values compared to untreated samples. (**E**) The DNAzyme-LAMP assay (60 min) differentiates strains, where PMAxx-treated susceptible strains exhibit a “signal-off” readout, while resistant strains display a “signal-on” readout.

In this study, we addressed the critical need for a rapid AST assay that combines short antibiotic exposure time with a simple readout, without relying on complex PCR-based nucleic acid detection methods. We developed and evaluated a new phenotypic AST method by combining PMAxx for live-dead differentiation and cDDLAMP for sensitive detection of *E. coli*. The method is named PMAxx dye-assisted colorimetric dual DNAzyme-LAMP (PD-cDDLAMP), and the principle of the method is depicted in Fig. 1. This approach addresses three key challenges: first, it enhances suppression of nucleic acid amplification from antibiotic-susceptible bacteria through selective binding of the photoreactive DNA-crosslinking dye PMAxx to the DNA of membrane-compromised cells after only 30 min of antibiotic exposure; second, it employs rapid isothermal amplification; and third, it uses a user-friendly colorimetric DNAzyme-LAMP readout system, enabling reliable discrimination between antibiotic-resistant ("signal-on") and antibiotic-susceptible ("signal-off") bacteria.

## MATERIALS AND METHODS

### Microorganisms and culturing

Reference strains of *E. coli* (ATCC 25922 and K-12) were obtained from the American Type Culture Collection (ATCC, Manassas, VA, USA) and used as antibiotic-susceptible control strains (27, 28). Additionally, eight previously reported *E. coli* clinical isolates (RM-6, RM-4, AG-3, AG-1, DM-2, DM-15, CD-Notag, and CD-G11) were provided by the Department of Animal Science at the University of Tennessee (29). Two additional *E. coli* clinical isolates (22-4401 and 22-4183) were provided by the Clinical Bacteriology and Mycology Laboratory (Biomedical and Diagnostic Sciences, College of Veterinary Medicine, University of Tennessee). All clinical isolates were identified using matrix-assisted laser desorption ionization time-of-flight mass spectrometry (MALDI-TOF MS) (Bruker Daltonics, Billerica, MA, USA) at the University of Tennessee’s Diagnostic Bacteriology and Mycology Laboratory.

The isolates were cultured from −80°C glycerol stocks in 5 mL of Mueller–Hinton broth (MHB) (Thermo Scientific Chemicals) for 18 h at 37°C in a shaking incubator at 225 rpm. The overnight bacterial culture was diluted in fresh MHB to achieve an optical density (OD) equivalent to a 0.5 McFarland standard, corresponding to approximately 10^8^ colony-forming units (CFU) per mL.

### Phenotypic conventional antimicrobial susceptibility testing

AST was performed previously using the broth microdilution method with commercially available 96-well microtiter plates (Sensitire CMV4AGNF, Thermo Fisher Scientific) (29). The minimum inhibitory concentrations (MIC) of ampicillin (AMP) and tetracycline (TET) were determined following the manufacturer’s protocol and the Clinical and Laboratory Standards Institute (CLSI) M100 guidelines for Enterobacteriaceae (30). The detailed antimicrobial susceptibility phenotypes of the clinical isolates were determined previously (29, 31) and are summarized in Table S1.

### Cell preparation and antibiotic treatment

AMP and TET were purchased from Thermo Scientific Chemicals. These antibiotics were selected due to their frequent use in mastitis treatment (32, 33). Antibiotic stock solutions (10 mg/mL) were prepared by dissolving 10 mg of each antibiotic in dimethyl sulfoxide (DMSO) to a final volume of 1 mL. The stock solutions were aliquoted and stored at −20°C. Working concentrations of the antibiotics were prepared by diluting the stock solutions in fresh MHB. The antibiotic concentrations used (32 μg/mL for AMP and 16 μg/mL for TET) were based on clinical breakpoint concentrations defined by the CLSI (34) and previous studies (4, 14, 16, 18, 28, 35–39).

To assess antibiotic susceptibility based on the NA amplification, bacterial suspensions were adjusted to 10^7^ CFU/mL (18). A 100 μL aliquot of each bacterial suspension was mixed with 100 μL of AMP or TET to achieve final concentrations of 16 μg/mL or 8 μg/mL, respectively. The mixtures were incubated at 37°C for 30 min in a heat block (17).

The experimental system included the susceptible reference strains (ATCC 25922 and K-12) together with clinical *E. coli* isolates exhibiting different susceptibility phenotypes to ampicillin and tetracycline, as determined by conventional broth microdilution AST (Table S1).

Positive (“Live”) and negative (“Dead”) controls were prepared by mixing 100 μL of bacterial suspensions with 100 μL of MHB without antibiotics. The dead control was prepared by heating the bacterial suspension at 95°C for 5 min to lyse the cells, followed by cooling on ice for 3 min.

For each sample, two sets of treatments were prepared: one for treatment with a viability dye (PMAxx-treated cells) and the other as a control without the dye (PMAxx-untreated cells).

### Viability discrimination assay

PMAxx DNA-intercalating dye (Biotium) was used to distinguish between live and dead cells, as well as between susceptible and resistant strains. The manufacturer’s protocol was followed with slight modifications. A 20 mM PMAxx stock solution was diluted with nuclease-free water (Sigma-Aldrich, St. Louis, MO) to a final concentration of 5 mM under minimal light.

5× PMA Enhancer (Biotium) was diluted to a 1× final concentration by adding 50 μL to 200 μL of bacterial suspension mixed with tetracycline. PMAxx stock was added to achieve a final concentration of 25 μM (e.g., 1 μL of 5 mM stock in 200 μL or 1.25 μL in 250 μL when using the enhancer). The mixture was incubated in the dark at room temperature for 10 min with shaking at 180 rpm. Tubes were then placed on ice 20 cm from a 500 W halogen lamp for 10 min to photoactivate PMAxx and cross-link it with free DNA.

DNA was extracted from pelleted bacterial cells by centrifugation at 8,000 × g for 10 min. The supernatant was discarded, and the cell pellet was resuspended in 200 μL of nuclease-free water (Sigma-Aldrich). The suspension was heated at 95°C for 15 min in a heat block (Thermo Fisher Scientific) and centrifuged at 12,000 × g for 2 min to sediment cell debris. The supernatant containing extracted DNA was used as the genomic DNA (gDNA) template for the cDDLAMP assay and viability quantitative PCR (qPCR).

### cDDLAMP assay

The DNAzyme-LAMP reaction targeting the *phoA* gene was performed using a previously validated protocol (25) with minor modifications. The reaction was conducted in a final volume of 10 μL containing 0.8 μM inner primers (FIP-rcEAD2 and BIP-rc Dz-00), 0.2 μM outer primers (F3 and B3), 0.4 μM loop primers (LF and LB), 5 μL WarmStart LAMP 2× Master Mix, 3 μL nuclease-free water, and 1 μL genomic DNA extract. The reaction mixture was incubated at 65°C for 60 min, followed by enzyme inactivation at 95°C for 2 min. A no-template control (NTC) was included using water instead of genomic DNA.

The DNAzyme-LAMP product (10 μL) was mixed with 0.75 μL of 200 μM hemin (MP Biomedicals) in DMSO and incubated at 95°C for 5 min. The mixture (10 μL) was then combined with 50 μL of tetramethylbenzidine (TMB) substrate (Pierce TMB Substrate Kit, Thermo Fisher Scientific) and incubated at room temperature for 1 min. OD at 655 nm was measured using a plate reader (Model 680, Bio-Rad). The signal-to-noise ratio (S/N) was calculated as follows:


S/N=(OD sample−OD blank)/(OD NTC−OD blank)

### Viability quantitative PCR

Real-time qPCR was performed using previously validated F3 and B3 primers targeting the *phoA* gene (25). The reaction was conducted in a final volume of 15 μL containing 0.6 μL of each primer (4 μM), 7.5 μL PowerUp SYBR Master Mix (2×, Applied Biosystems), 4.8 μL nuclease-free water, and 1.5 μL genomic DNA extract. Amplification was performed using a QuantStudio Real-Time PCR System with the following cycling parameters: initial denaturation at 95°C for 1 min, followed by 40 cycles of denaturation at 95°C for 15 s, annealing at 50°C for 30 s, and extension at 60°C for 1 min. A NTC was included.

ΔCt values were calculated according to the manufacturer’s instructions as follows:


ΔCt live untreated control=Ct(live, PMAxxtreated)−Ct(live, untreated)

ΔCt dead untreated control=Ct(dead, PMAxxtreated)−Ct(dead, untreated)

ΔCt antibiotic−treated samples=Ct(sample, PMAxxtreated)−Ct(sample, untreated)

### Milk spiking and pretreatment with proteinase K

Fifty microliters of viable *E. coli* ATCC 25922 (AMP, TET-sensitive) and clinical strain 22-4183 (AMP, TET-resistant) were added to 450 μL of pasteurized milk to achieve a final concentration of 10^7^ CFU/mL. Dead control cells were prepared as described earlier. Milk samples were subjected to proteinase K treatment as previously mentioned (40, 41) with little modification. Milk samples were treated with 12.5 μL of proteinase K (20 mg/mL) at 37°C for 15 min to digest milk proteins and avoid the use of 56°C that may cause mild heat treatment affecting the bacterial cell wall integrity. Samples were centrifuged at 8,000 × g for 6 min, and the supernatant was discarded. The pellet was washed once with 200 μL of 1 × PBS and centrifuged at 12,000 × g for 2 min. The pellet was resuspended in 200 μL of MHB with or without antibiotics to achieve final concentrations of 16 μg/mL (AMP) or 8 μg/mL (TET). Antibiotic exposure, PMAxx treatment, DNA extraction, cDDLAMP, and viability qPCR were performed as described above.

### Data analysis

A two-way ANOVA was performed to assess significant differences in the signal-to-noise ratio (S/N) of the PD-cDDLAMP between live and dead controls, as well as between drug-sensitive and drug-resistant strains following treatment with PMAxx, enhancer, or a combination of both. Post hoc tests were conducted using Tukey’s honestly significant difference method. Data represent two independent experiments with two replicates each (*n* = 4), with error bars indicating standard deviations. A *P*-value < 0.05 was considered statistically significant.

Susceptibility/resistance thresholds for SNR were established based on the positive cutoff value, calculated as the mean of the NTC plus two standard deviations (95% CI)(42). All analyses were performed using JMP Pro, Version 17 (64-bit), and graphs were prepared using GraphPad Prism (version 8, GraphPad Software Inc.). Schematics were created using BioRender.com.

A confusion matrix was used to display true positive (TP), true negative (TN), false positive (FP), and false negative (FN) counts for each antibiotic or combined model. The following metrics were calculated as previously mentioned (43, 44):

- Sensitivity (True Positive Rate, TPR) = TP/(TP+FN)
- Specificity (True Negative Rate, TNR) = TN/(FP+TN)
- False Positive Rate (FPR) = FP/(FP+TN)
- False Negative Rate (FNR) = FN/(FN+TP)
- Accuracy (ACC) = (TP+TN)/(TP+TN+FP+FN)

## RESULTS

### Effect of pretreatments with PMAxx and enhancer on the evaluation of *E. coli* viability

**Fig 2:**
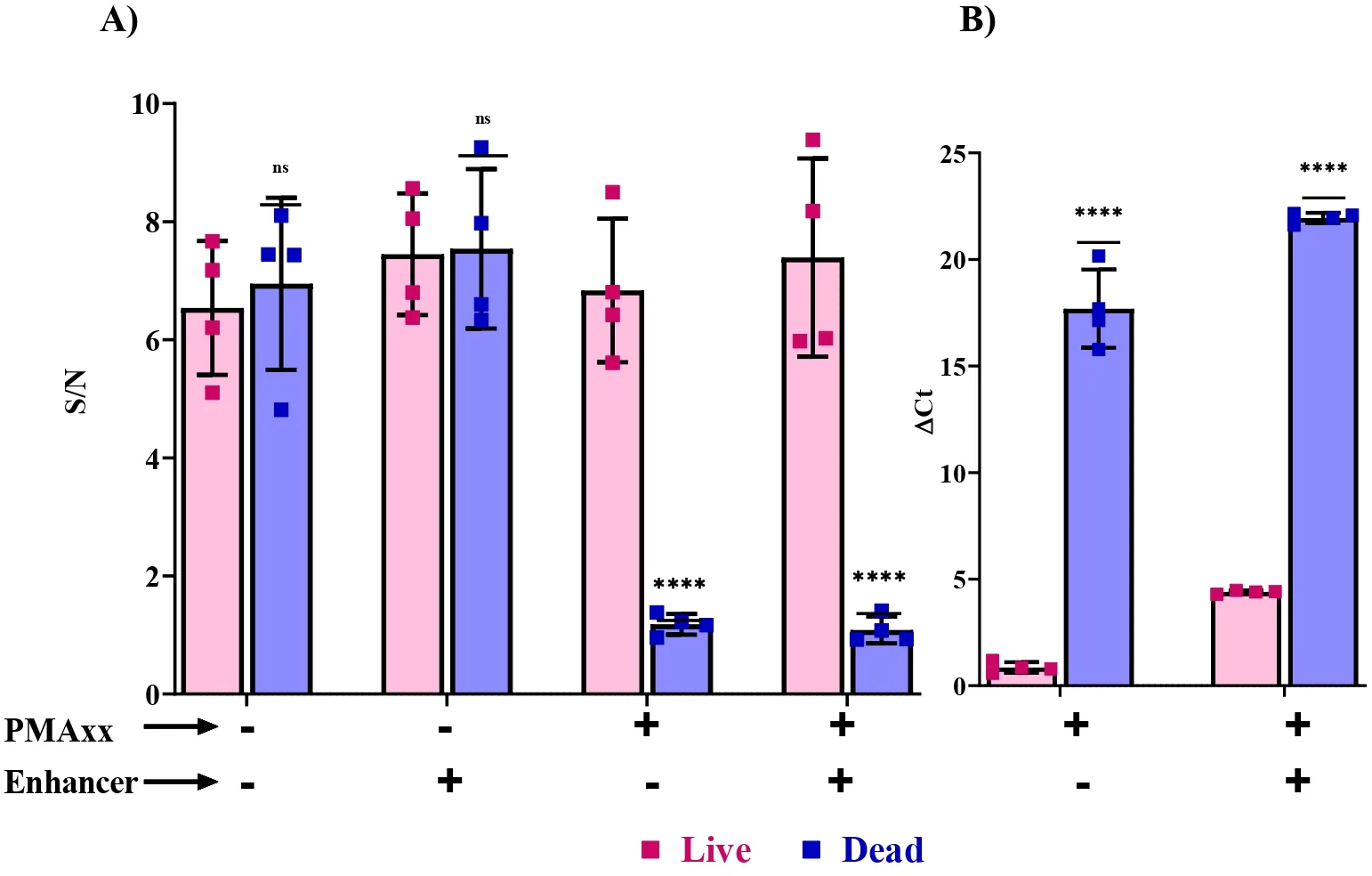
Impact of PMAxx and PMA Enhancer (Enhancer) on *E. coli* cell viability. (**A**) Viability of *E. coli* cells was assessed using PD-cDDLAMP with a signal-off detection approach following treatment with PMAxx, Enhancer, a combination of both, or no treatment. The signal-to-noise ratio (S/N) was calculated as the ratio of the OD (optical density at 655 nm) for the positive sample to the no-template control, with buffer background OD subtracted from both values. (**B**) Comparison of the ∆Ct values in qPCR between PMAxx alone, combined PMAxx and enhancer treatment, and untreated genomic DNA samples. ∆Ct is defined as the Ct difference between treated and untreated samples for both live and dead *E. coli* cells. Raw Ct values are provided in Table S2. Error bars represent the standard error of two independent experiments, each with four replicates (*n* = 4). Statistical analysis is denoted as ****P < 0.0001, ns, *P* > 0.05.

To assess the effect of PMAxx reagents on distinguishing dead from live bacteria, DNA was extracted from live and dead *E. coli* and amplified using DNAzyme-LAMP following treatment with PMAxx, enhancer, both PMAxx and enhancer, or with no treatment. As expected, there was no significant difference between nontreated dead DNA samples and live DNA samples (ns, *P* = 0.9995) (Fig. 2A; Fig. S1). Similarly, treating dead DNA samples with the enhancer alone did not result in a significant distinction from live DNA samples (ns, *P* = 1) (Fig. 2A; Fig. S1), as this treatment alone does not discriminate between live and dead cells.

In contrast, PMAxx was highly effective in differentiating live from dead bacteria, essentially “switching off” the signal from dead bacteria (****, *P* < 0.0001) (Fig. 2A; Fig. S1). This differentiation was observed when PMAxx was used with or without the enhancer, with no significant difference in effectiveness between the two treatment methods on dead cells (ns, *P* = 1) (Fig. 2A; Fig. S1). None of the treatments (PMAxx, enhancer, or their combination) significantly impacted the signals in live cell samples compared to those of untreated live samples (ns, *P* = 0.9999, 0.9477, 0.9622, respectively) (Fig. 2A; Fig. S1).

To further verify whether PMAxx effectively inhibited amplification of dead cell DNA, viability qPCR was used to validate the DNAzyme-LAMP results by calculating the delta Ct (∆Ct) between treated and untreated samples for both live and dead *E. coli*. In qPCR, dead cell DNA showed delayed amplification and higher ∆Ct values than those live cells when treated with either PMAxx alone or with both PMAxx and enhancer (****, *P* < 0.0001) (Fig. 2B; Table S2).

### Effect of using an enhancer with PMAxx on the evaluation of *E. coli* viability under AMP or TET exposure

Both the antibiotic-susceptible *E. coli* reference strain (ATCC 25922) and resistant strain (22-4183) were exposed to AMP or TET at CLSI breakpoint concentrations (16 and 8 μg/mL, respectively) for 30 min. Following antibiotic treatment, the samples were treated with PMAxx alone or with both PMAxx and enhancer. Genomic DNA samples were extracted and amplified using DNAzyme-LAMP. PMAxx-treated live and dead cells were used as controls.

**Fig 3:**
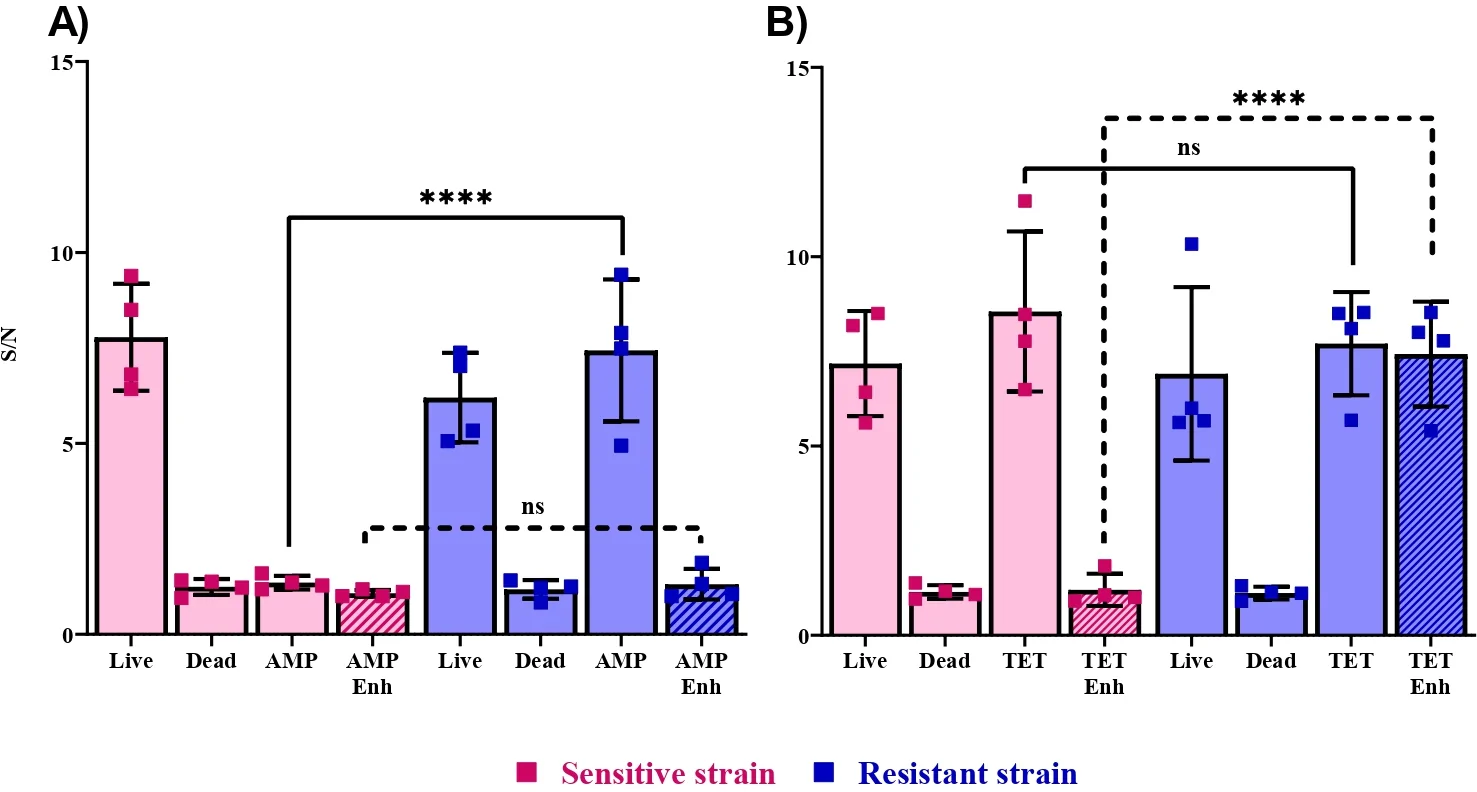
Impact of PMAxx and PMA Enhancer (Enh) on *E. coli* susceptibility to AMP and TET. Signal-on and off detection approaches for antibiotic-susceptible and -resistant *E. coli* strains upon exposure to AMP (**A**) and TET (**B**) were evaluated using PD-cDDLAMP. Cells were treated with either PMAxx alone or PMAxx plus the enhancer. Heat-lysed dead and live cells served as controls. The signal-to-noise ratio (S/N) was determined by calculating the ratio of the OD (optical density at 655 nm) for each positive sample relative to the no-template control, with buffer background OD subtracted from both values. Error bars represent the standard error from two independent experiments, each with four replicates (*n* = 4). Statistical analysis is denoted as ****P < 0.0001, ns, *P* > 0.05.

The DNAzyme-LAMP reaction was inhibited in the susceptible strain regardless of the enhancer treatment (ns, *P* = 1). On the other hand, the reaction was not inhibited in the resistant strain in the absence of enhancer, allowing a significant differentiation between susceptible and resistant strains (****, *P* < 0.0001) (Fig. 3A; Fig. S2). In the resistant strain, the DNAzyme-LAMP reaction was also inhibited in the presence of enhancer, and the signal was indistinguishable from that of heat-lysed dead cells (ns, *P* = 1) and from AMP-exposed susceptible *E. coli* (ns, *P* = 0.9999), indicating that PMAxx alone, without the enhancer, effectively penetrated the AMP-compromised membrane, enabling clear differentiation between AMP-susceptible and resistant *E. coli* (****, *P* < 0.0001) (Fig. 3A; Fig. S2).

In the TET susceptibility assay, as TET inhibits bacterial growth by binding to ribosomes and stopping protein synthesis, the susceptible *E. coli* strains may retain intact membranes that limit permeability to PMAxx dyes. When only PMAxx was used, there was no significant difference between TET-exposed resistant and susceptible *E. coli* samples (ns, *P* = 0.9872) (Fig. 3B; Fig. S2). However, the addition of the PMA enhancer for Gram-negative bacteria significantly improved PMAxx’s effectiveness under TET exposure, which is less disruptive to bacterial membranes. A substantially higher signal was observed for tetracycline-exposed resistant *E. coli*, while the signal for TET-exposed susceptible *E. coli* was effectively “switched off” (****, *P* < 0.0001) (Fig. 3B; Fig. S2).

### Signal-to-noise ratio and qPCR ΔCt values in antibiotic-exposed *E. coli* strains with and without PMAxx

We compared the signal-to-noise ratio (S/N) for AMP- and TET-exposed *E. coli* cells, as well as heat-lysed dead and live cells, across both antibiotic-resistant and susceptible strains. For each sample, treatments were conducted both with (+) and without (−) PMAxx.

**Fig 4:**
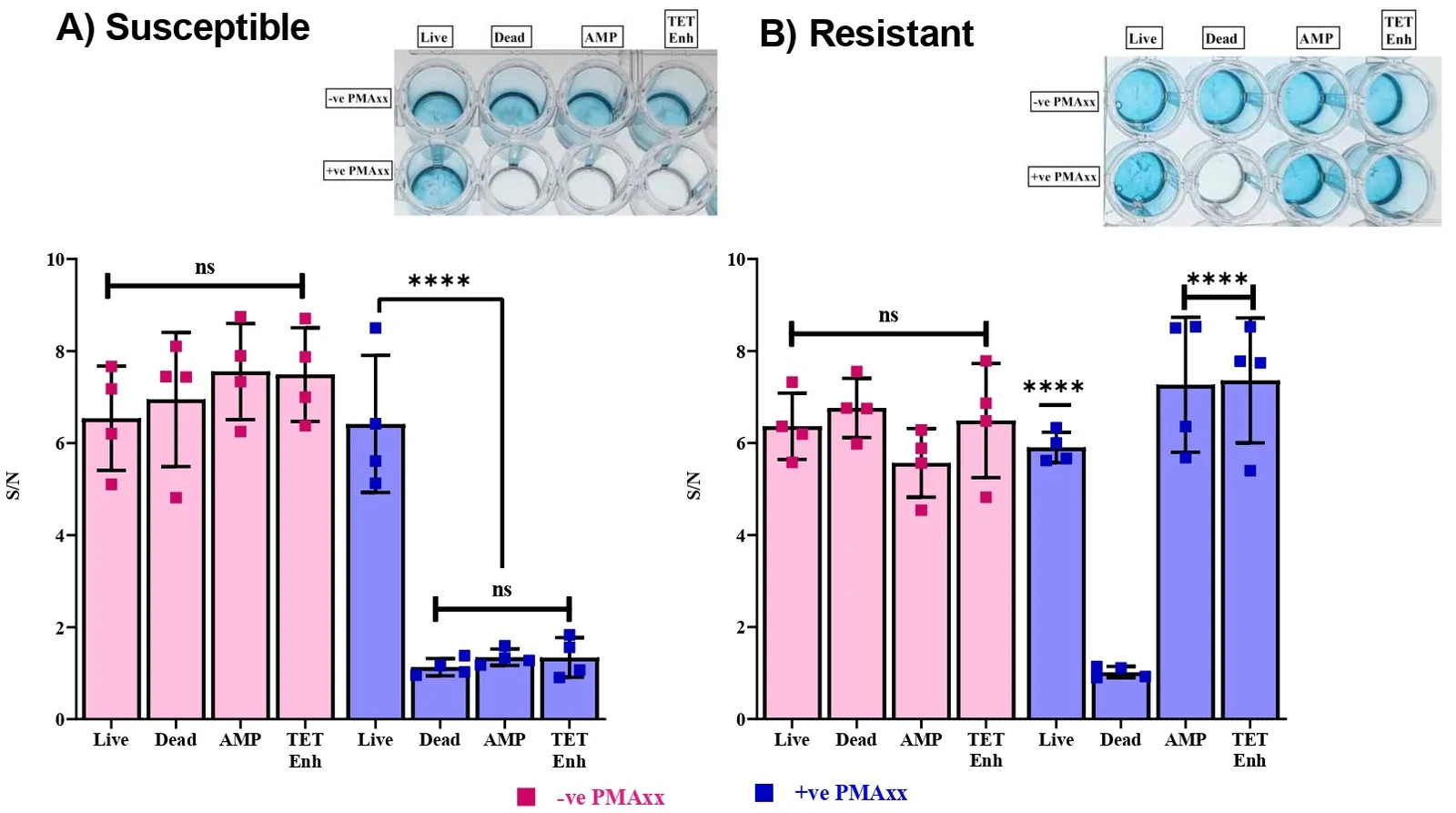
Comparison of signal-to-noise ratio (S/N) in Antibiotic-Exposed, Dead, and Live *E. coli* cells treated with PMAxx. The signal-to-noise ratio (S/N) was measured for both antibiotic-susceptible (**A**) and -resistant (**B**) *E. coli* strains following exposure to AMP or TET, as well as for heat-lysed dead and live cells. Each sample was tested with (+) and without (−) PMAxx treatment. An inset photograph illustrates PMAxx-treated susceptible strains displaying a “signal-off” response, while resistant strains show a “signal-on” response. SNR was calculated as the ratio of the OD (optical density at 655 nm) of each positive sample relative to the no-template control, with buffer background OD subtracted. Error bars represent the standard error from two independent experiments, each with four replicates (*n* = 4). Statistical analysis is denoted as ****P < 0.0001, ns, *P* > 0.05.

In general, the PMAxx(−) samples showed no difference in signal-to-noise ratio across the AMP- and TET-exposed *E. coli* and dead and live cells (ns, *P* > 0.6) (Fig. 4).

In the PMAxx(+) samples of the susceptible strain, no significant difference was observed between AMP-exposed, TET-exposed, and dead cells (ns, *P* > 0.6), all of which showed a “signal-off” profile, allowing clear distinction from live cells (****, *P* < 0.0001) (Fig. 4A). In the resistant strain, no significant difference was found among AMP-exposed, TET-exposed, and live cells (ns, *P* > 0.3), all of which showed a “signal-on” profile, clearly distinguishing them from dead cells (****, *P* < 0.0001) (Fig. 4B).

**Fig 5:**
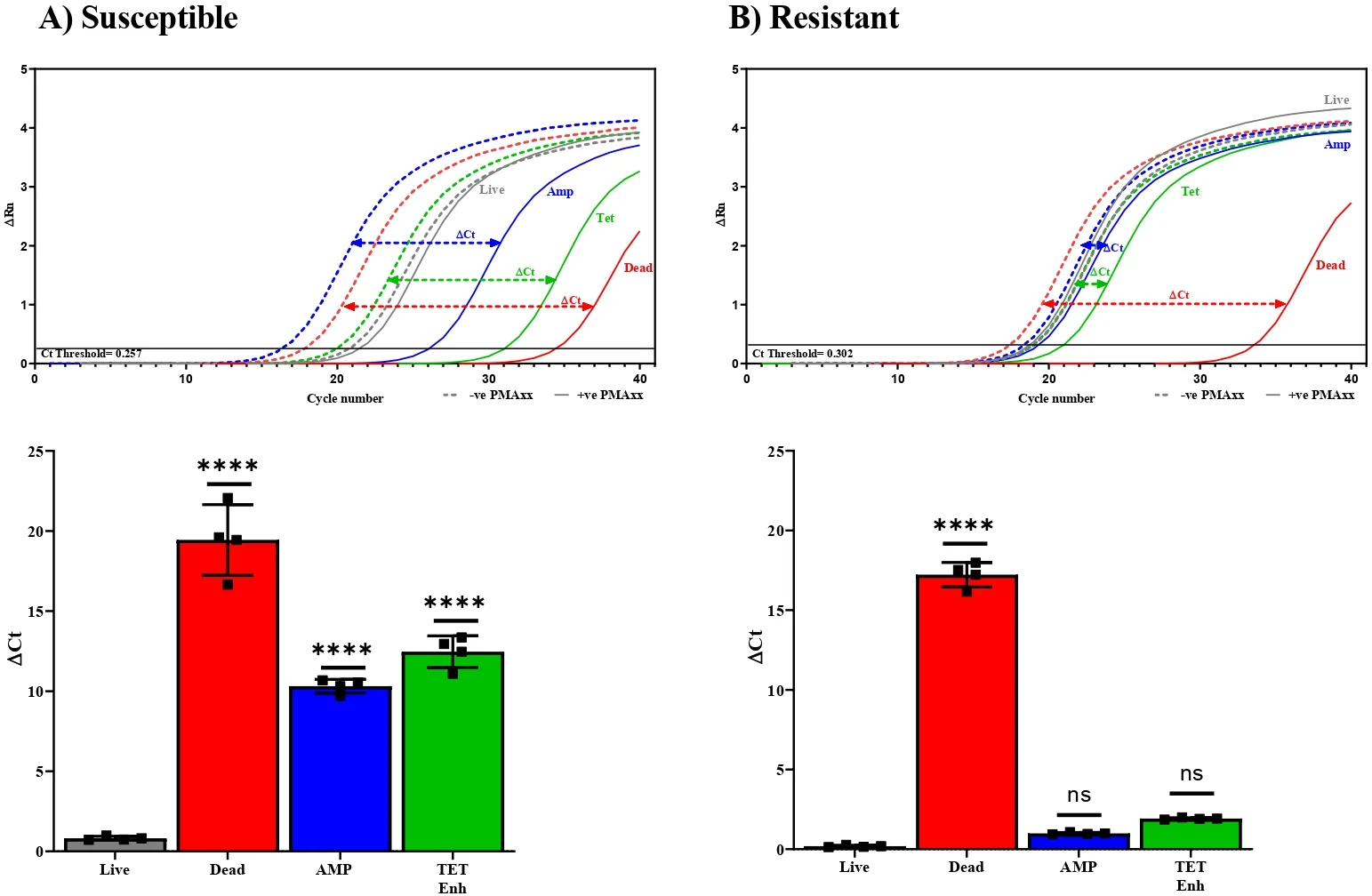
Quantitative PCR analysis of ΔCt Values for PMAxx-treated and untreated genomic DNA from *E. coli* strains exposed to AMP and TET, comparing dead (heat-lysed) and live cells in both antibiotic-susceptible (**A**) and antibiotic-resistant (**B**) strains. The insert shows representative PCR amplification plots of the same samples, displaying normalized fluorescence. ΔCt values are calculated as the difference in Ct between PMAxx-treated and untreated samples. Raw Ct values are provided in Table S3. Error bars represent the standard error from two independent experiments, each performed with four replicates (*n* = 4). Statistical analysis is denoted as ****P < 0.0001, ns, *P* > 0.05.

qPCR Ct values were measured for AMP-exposed and TET-exposed *E. coli*, heat-lysed dead, and live cells across both resistant and susceptible strains, with and without PMAxx. ΔCt values, calculated as the difference in Ct between PMAxx-treated and untreated samples, were analyzed. There was a significant difference in ΔCt between PMAxx-treated dead and live cells in both susceptible and resistant *E. coli* strains (****, *P* < 0.0001) (Fig. 5; Table S3). A significantly higher ΔCt value was observed in PMAxx(+) AMP- and TET-treated susceptible *E. coli* compared to live controls (****, *P* < 0.0001) (Fig. 5A; Table S3). In contrast, PMAxx(+) AMP- and TET-treated resistant *E. coli* showed lower ΔCt values, which were not significantly different from live controls (ns, *P* = 0.9099 and 0.1734, respectively) (Fig. 5B; Table S3).

### Performance of PD-cDDLAMP in detecting AMP and TET resistance

**Fig 6:**
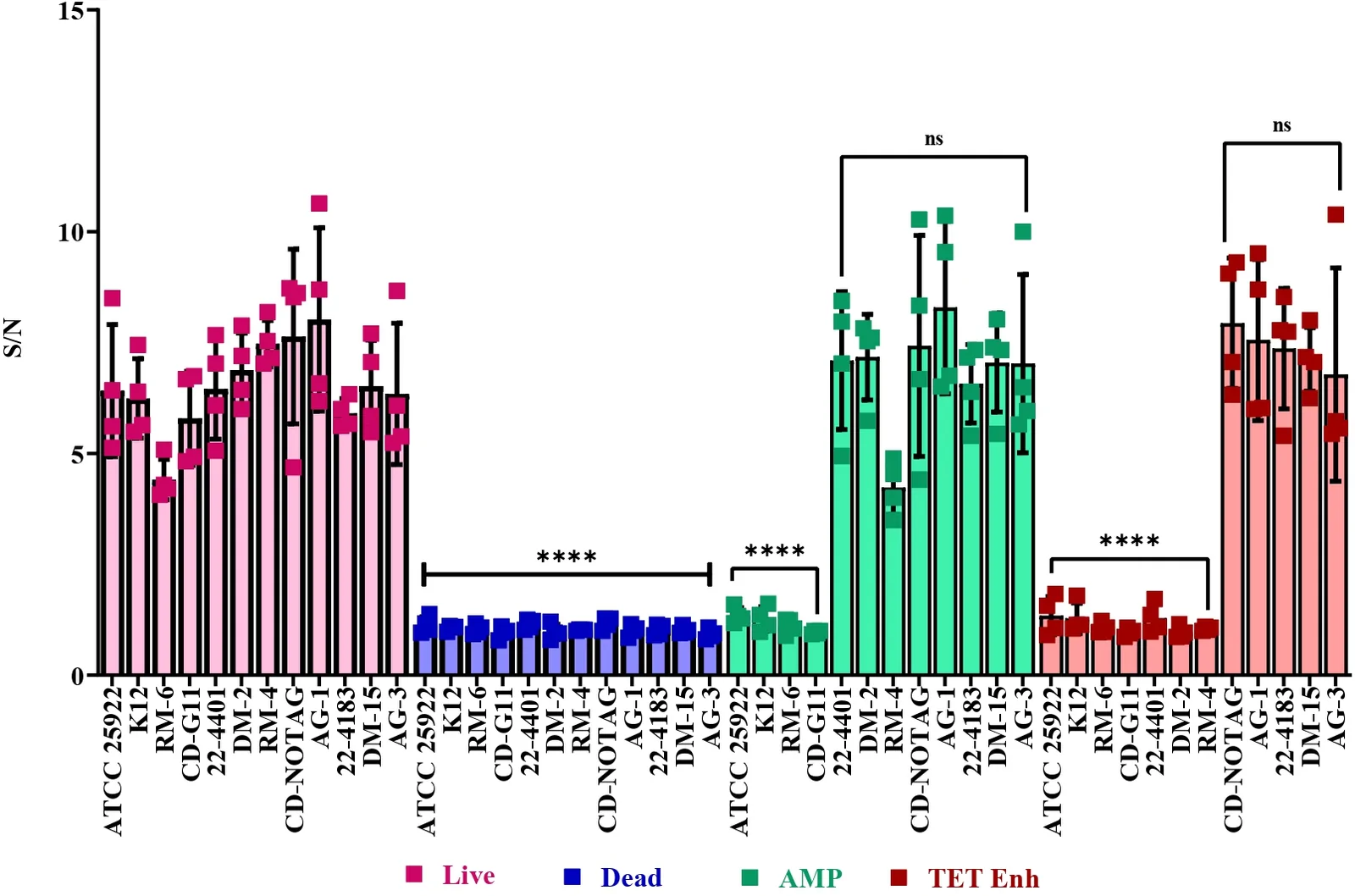
Rapid discrimination of antibiotic-susceptible and resistant *E. coli* using PD-cDDLAMP. S/N was calculated as the ratio of the OD (optical density at 655 nm) of each positive sample relative to the no-template control, with buffer background OD subtracted. The mean and standard error of the mean are shown for two independent experiments, each performed with four replicates (*n* = 4) of each clinical isolate. Statistical analysis is denoted as ****, *P* < 0.0001, ns, *P* > 0.05.

**Fig 7:**
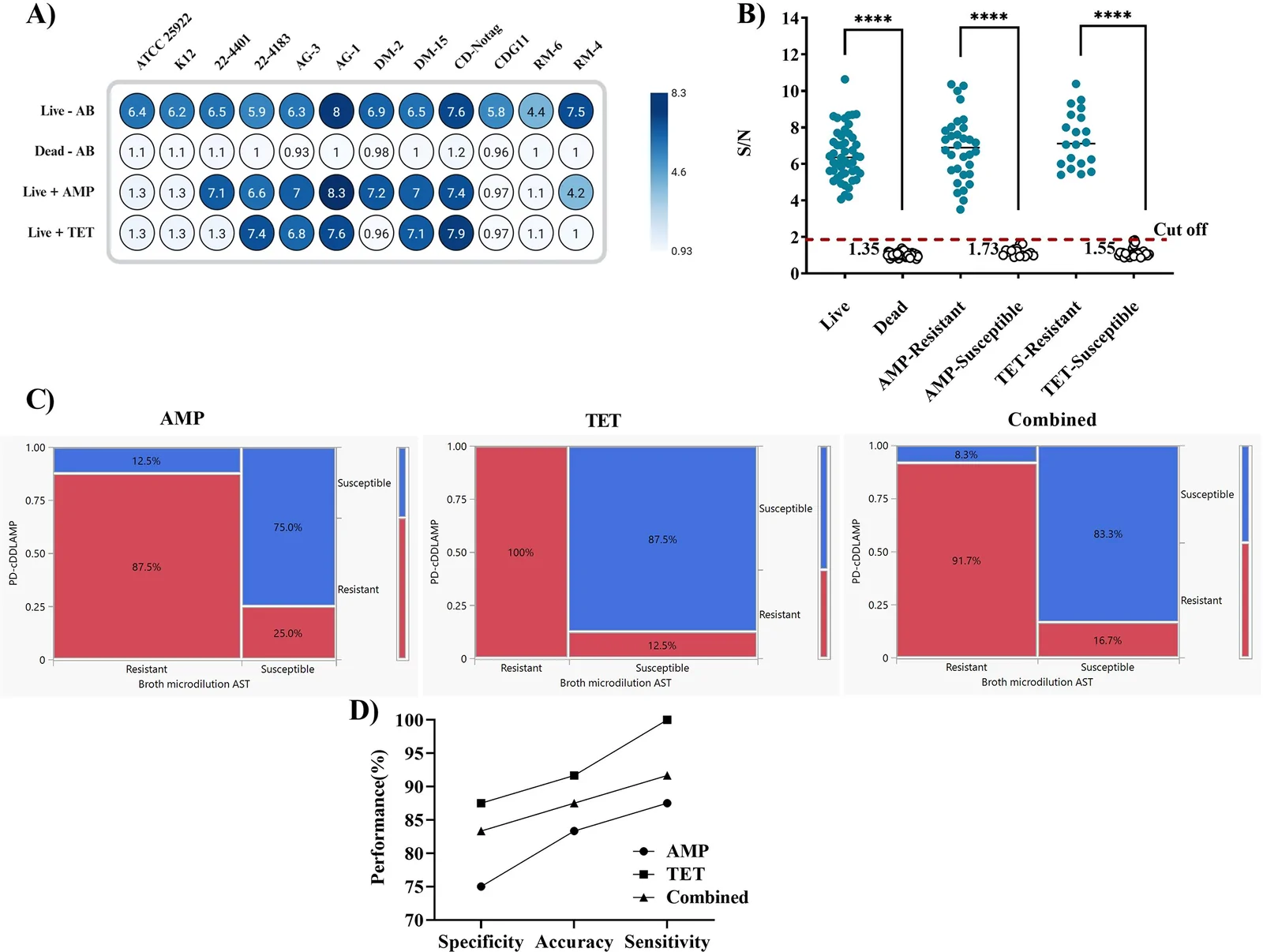
Performance of the PD-cDDLAMP antimicrobial susceptibility test (AST) for AMP and TET. (**A**) Heatmap displaying the signal-to-noise (S/N) ratios obtained for different bacterial strains under various conditions: live without antibiotics (Live - AB), heat-killed without antibiotics (Dead - AB), live with AMP (Live + AMP), and live with TET (Live + TET). Darker blue shades indicate higher S/N values.(**B**) Scatter plot showing signal-on (blue dots), and signal-off (white dots) approaches illustrating the distribution of S/N values for different bacterial conditions, including live, dead, AMP-resistant, AMP-susceptible, TET-resistant, and TET-susceptible groups. The red dashed line represents the susceptibility/resistance thresholds of S/N > 1.35, 1.73, and 1.55, respectively, established based on the positive cutoff value calculated as the mean of the no-template control (NTC) plus two standard deviations (95% CI). Raw data across a range of clinical *E. coli* isolates and two ATCC reference *E. coli* strains are shown, and statistical significance is indicated (****, *P* < 0.0001). (**C**) Agreement plots showing comparison analysis of PD-cDDLAMP (y-axis) versus broth microdilution AST (x-axis) for AMP and TET, as well as a combined classification for both antibiotics. Each mosaic plot shows the proportional distribution of isolates classified as resistant or susceptible by both methods. (**D**) Performance evaluation of the classification model based on specificity, accuracy, and sensitivity for AMP (circles), TET (squares), and combined (triangles) data sets.

The performance of the rapid PD-cDDLAMP method for AST was assessed across 10 *clinical E. coli* strains, which had been previously classified using standard AST methods. Additionally, two ATCC reference *E. coli* strains were included in the evaluation. The results, displayed in Fig. 6, Fig. S3, and Fig. 7A, reveal a clear separation between AMP- and TET-susceptible and -resistant strains, alongside a distinction between live and dead bacterial cells (****, *P* < 0.0001, Fig. 6). Additionally, there was no statistically significant difference in signal output between live *E. coli* cells and AMP- or TET-resistant strains (ns, *P* = 0.7591 and 0.0639, respectively; Fig. 6). The scatter plot illustrated the separation with defined thresholds for each condition, with cutoff values of 1.73, 1.55, and 1.35, respectively (Fig. 7B), supporting the reliability of the proposed AST in distinguishing these categories.

Our results demonstrated a high degree of consistency between the proposed rapid PD-cDDLAMP assay and the conventional broth microdilution AST, with only three discrepancies among 24 total AST data points. Specifically, sample #CD-NOTAG was misclassified as resistant to AMP, sample #RM-6 was misclassified as susceptible to AMP, and sample #CD-NOTAG was misclassified as resistant to TET.

Agreement plots (Fig. 7C) illustrate the comparison analysis of PD-cDDLAMP (y-axis) versus broth microdilution AST (x-axis) for AMP and TET, as well as a combined classification for both antibiotics. These mosaic plots compare the predicted susceptibility/resistance profiles obtained by PD-cDDLAMP with the reference (true) profiles determined by broth microdilution AST, where the bars represent the proportional distribution of isolates classified as resistant or susceptible by both methods. For AMP, classification outcomes included 87.5% true-resistant (7/8), 75% true-susceptible (3/4), 25% false-resistant (1/4), and 12.5% false-susceptible (1/8). For TET, results showed 100% true-resistant (4/4), 87.5% true-susceptible (7/8), 12.5% false-resistant (1/8), and no false-susceptible results. The combined classification for both antibiotics demonstrated 91.67% true-resistant (11/12), 83.3% true-susceptible (10/12), 16.67% false-resistant (2/12), and 8.3% false-susceptible (1/12).

Quantitatively, the PD-cDDLAMP achieved a sensitivity, specificity, and accuracy of 87.5%, 75%, and 83.33% for AMP detection, correctly identifying 3 of 4 susceptible and 7 of 8 resistant strains (Fig. 7D). For TET, these values were even higher—100%, 87.5%, and 91.67%, respectively—accurately classifying all seven susceptible strains and 4 of 5 resistant strains (Fig. 7D). When AMP and TET were analyzed in combination, the assay achieved a specificity of 83.33%, sensitivity of 91.67%, and an overall accuracy of 87.5% (Fig. 7D). These results indicate strong categorical agreement between PD-cDDLAMP and the standard AST method, underscoring the assay’s potential as a rapid, reliable tool for antimicrobial susceptibility testing.

### Validation of PD-cDDLAMP for AST in milk samples and comparison with qPCR ΔCt analysis

**Fig 8:**
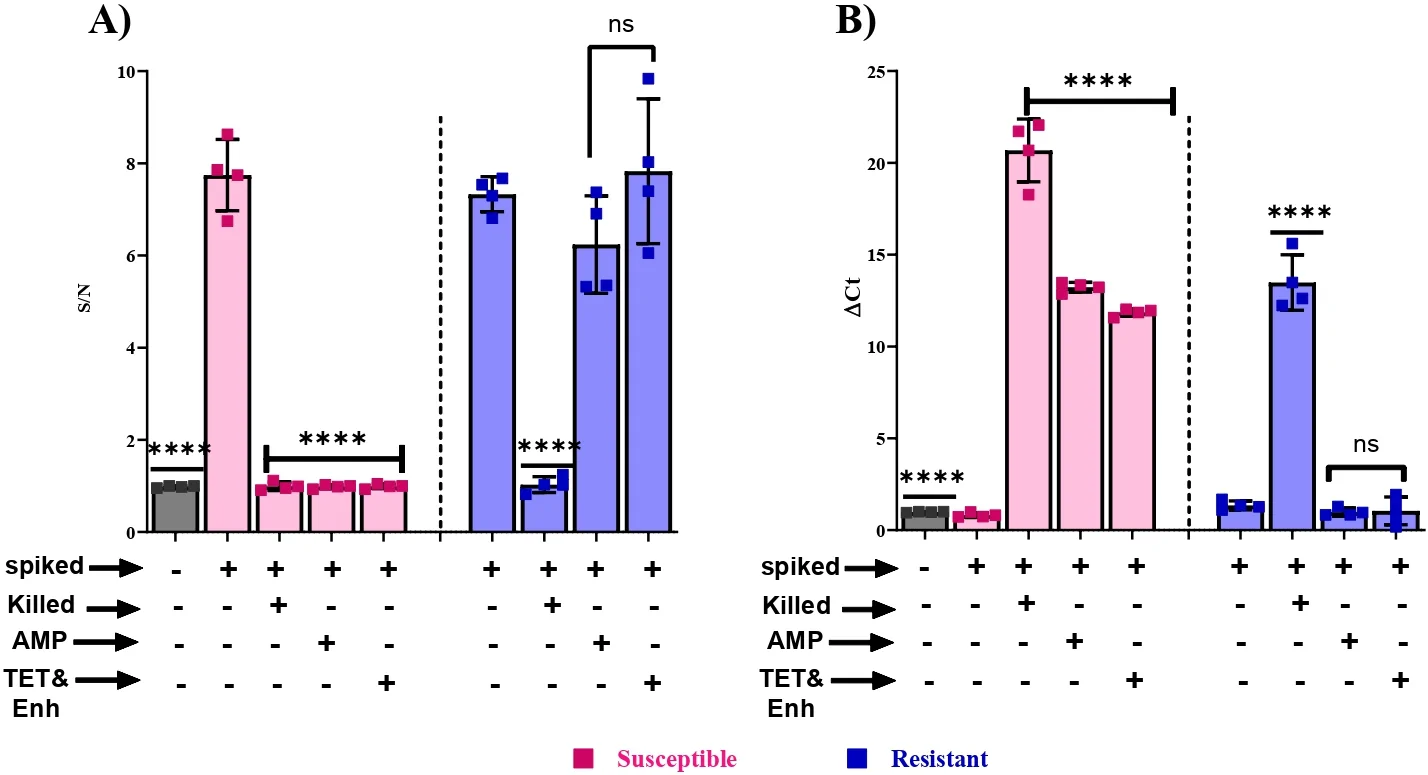
Rapid antimicrobial susceptibility testing (AST) of *E. coli* from spiked milk samples using the PD-cDDLAMP method. The PD-cDDLAMP method enables AST of *E. coli* from spiked milk samples within 2 h. Following proteinase K purification (15 min), the cell pellet is incubated with an antibiotic for 30 min to assess drug sensitivity by measuring changes in bacterial viability through PMAxx treatment. The assay provides susceptibility information for AMP and TET, aiding in antibiotic prescription decisions. (**A**) A clear signal-on/off approach differentiates resistant *E. coli* strains from susceptible strains. S/N was calculated as the ratio of the OD (optical density at 655 nm) of each positive sample relative to the no-template control, with buffer background OD subtracted. (**B**) Validation of the PD-cDDLAMP method through qPCR analysis of ΔCt values in PMAxx-treated spiked milk samples. ΔCt values are calculated as the difference in Ct between PMAxx-treated and untreated samples. Raw Ct values are provided in Table S4. The mean and standard error of the mean are shown for two independent experiments, each with four replicates (*n* = 4) of spiked and unspiked control samples. Statistical analysis is denoted as ****, *P* < 0.0001, ns, *P* > 0.05.

To validate the effectiveness of PD-cDDLAMP in pathogen-specific antimicrobial detection, two milk samples were spiked with resistant and susceptible *E. coli* strains at a concentration of 1.5 × 10^8^ CFU/mL. As shown in Fig. 1, the entire process was completed in less than 2.5 h, encompassing proteinase K digestion of milk proteins (15 min), antibiotic exposure (30 min), PMAxx application (20 min), and DNAzyme LAMP (60 min). Signal-to-noise ratio analysis revealed a significant difference in detecting live *E. coli* strains in spiked milk samples compared to unspiked controls (****, *P* < 0.0001) (Fig. 8A). A clear distinction was observed between live, dead, and AMP- and TET-susceptible *E. coli* strains (****, *P* < 0.0001) (Fig. 8A). Additionally, a significant difference was observed between live and dead resistant *E. coli* strains (****, *P* < 0.0001) (Fig. 8A). In contrast, there was no significant difference in signal between live, AMP-resistant, and TET-resistant *E. coli* strains (ns, *P* = 0.654 and 0.999, respectively) (Fig. 8A).

To further confirm the reliability of our PD-cDDLAMP approach for AST in milk samples, we compared it with qPCR ΔCt values. We prepared the same spiked milk samples as in Fig. 8A, along with control aliquots without PMAxx treatment. ΔCt values were calculated as previously described. A significant difference in ΔCt values was observed between PMAxx-treated dead and live cells in both susceptible and resistant *E. coli* strains (****, *P* < 0.0001) (Fig. 8B; Table S4). Notably, a significantly higher ΔCt was detected in PMAxx(+) AMP- and TET-treated susceptible *E. coli* compared to live controls (****, *P* < 0.0001) (Fig. 8B; Table S4). However, PMAxx(+) AMP- and TET-treated resistant *E. coli* showed lower ΔCt values, which were not significantly different from live controls (ns, *P* = 0.999) (Fig. 8B; Table S4).

## DISCUSSION

β-lactam antibiotics (including AMP) and TET are among the most widely prescribed drugs in dairy farming in the United States, primarily for managing mastitis and other common diseases (32, 33, 45). However, the emergence of high resistance levels to these antibiotics, including up to 99.1% resistance to AMP and 73.9% to TET in *E. coli* isolates (46, 47)*,* poses a significant threat to both animal and human health. This study highlights the urgent need for improved antibiotic stewardship and rapid AST to combat resistance in dairy farming.

Current NA-based phenotypic AST often requires up to 4 h of antibiotic exposure to generate measurable differences (9, 12, 14). When exposure time is reduced to 30 min, more sensitive detection methods such as qPCR, digital PCR, or digital LAMP are required to resolve minimal differences in nucleic acid abundance by assessing changes in the DNA released from the susceptible cells either to enhancer-mediated nuclease digestion or polymerase amplification (15, 16). Although effective, these methods increase instrumentation demands and reduce workflow simplicity, limiting their applicability in rapid testing scenarios (2). Similarly, other advanced AST platforms, including nanomotion-based systems, deep learning-assisted phenotyping, plasmonic nanosensors, and droplet-based chromogenic assays, reduce time-to-result but introduce multi-step processing and analytical complexity (8, 35, 48–50). Collectively, these approaches highlight a central unresolved challenge in current AST: achieving both rapid antibiotic exposure and simple, robust signal readout.

Mechanistically, antibiotic class-specific cellular responses further influence NA-based AST performance. Antibiotics targeting DNA replication (e.g., fluoroquinolones) rapidly alter nucleic acid dynamics, enabling shorter exposure times for detection (13, 51). In contrast, antibiotics such as β-lactams and tetracyclines act indirectly by targeting cell wall synthesis and protein synthesis, respectively (52, 53), which delays measurable changes in DNA replication. As a result, susceptibility has been shown to remain undetectable after short exposure using qPCR or dPCR when DNA extraction conditions allow genome replication to continue (16).

Within this context, PMA/PMAxx-based viability dyes provide a mechanistic bridge by selectively binding DNA in membrane-compromised cells and preventing downstream amplification (15, 21). This strategy has been successfully applied in microbial viability assessment and rapid NA-based AST, including *M. tuberculosis*, *N. gonorrhoeae*, and nanopore-based metagenomic profiling (17, 18, 23). However, existing implementations still rely on PCR-based quantification or complex workflows, and thus do not fully resolve limitations in readout simplicity and system integration.

To overcome these limitations, PD-cDDLAMP integrates PMAxx-mediated suppression of amplification from non-viable or membrane-compromised cells with a colorimetric dual DNAzyme-LAMP readout, thereby converting subtle viability differences into a visually interpretable signal, enabling reliable discrimination between antibiotic-resistant and susceptible bacteria after only 30 min of antibiotic exposure.

Importantly, the PMAxx-assisted PD-cDDLAMP assay determines phenotypic antimicrobial susceptibility rather than identifying specific resistance mechanisms. The assay is based on bacterial viability following antibiotic exposure. In ampicillin-susceptible isolates, ampicillin binds penicillin-binding proteins (PBPs), inhibits cell wall synthesis, and ultimately causes cell wall damage, loss of viability, and membrane compromise. These membrane alterations permit PMAxx to penetrate the cells and bind genomic DNA, thereby preventing subsequent amplification. In contrast, ampicillin-resistant cells remain viable following antibiotic exposure. Regardless of whether resistance is mediated by β-lactamase production, reduced outer membrane permeability, active efflux, altered PBPs, or other mechanisms, these resistance determinants ultimately prevent ampicillin from inducing lethal cell wall damage. Consequently, PMAxx is excluded from viable resistant cells, allowing their DNA to remain amplifiable and generate a positive colorimetric signal. Thus, the assay reports the overall phenotypic outcome of antibiotic exposure rather than distinguishing the underlying molecular mechanism of resistance.

The applicability of this workflow was demonstrated using both β-lactams (ampicillin) and tetracycline, two antibiotics with distinct mechanisms of action. Despite their different biological targets, both classes require longer exposure times in conventional NA-based AST due to indirect effects on DNA replication (16). PD-cDDLAMP successfully enables rapid detection under a unified 30-min exposure condition, demonstrating robustness across antibiotic classes with different cellular response dynamics.

Importantly, the PD-cDDLAMP assay was evaluated in milk as one of the most challenging clinical matrices for molecular diagnostics because of its complex composition, including high concentrations of fats, proteins, lactose, somatic cells, and other components. In addition, milk contains several amplification inhibitors, such as calcium ions, immunoglobulins, lactoferrin, casein, and residual antimicrobial compounds. These matrix-associated inhibitors complicate the direct detection of antimicrobial-resistant bacteria (54–56).

The bacterial burden in milk is highly variable and closely associated with the severity and stage of mastitis (57). Previous studies have reported that bacterial concentrations increase substantially during acute mastitis infections, with *E. coli* counts reaching approximately 10⁷ CFU/mL in severe clinical mastitis (58). These clinically relevant bacterial loads provide a practical context for the development and evaluation of rapid molecular phenotypic AST methods. In contrast, conventional culture-based AST, following CLSI guidelines, uses a standardized inoculum of 2–8 × 10⁵ CFU/mL but requires 16–24 h of antibiotic exposure to determine susceptibility (59). Rapid molecular phenotypic AST methods, including nanopore sequencing- and LAMP-based phenotypic AST platforms, typically employ higher bacterial concentrations, around 10⁷ CFU/mL, to detect early physiological or nucleic acid changes after short antibiotic exposure (14, 18), supporting the use of this concentration for proof-of-concept optimization of the PD-cDDLAMP assay. Thus, the selected inoculum represents both a clinically relevant bacterial burden observed during acute *E. coli* mastitis and a practical strategy for achieving rapid phenotypic antimicrobial susceptibility determination in a highly complex milk matrix.

In terms of analytical performance, PD-cDDLAMP achieved high sensitivity and overall accuracy, outperforming several previously reported rapid phenotypic AST platforms, including single-cell imaging and machine learning-based phenotyping approaches (48). While specificity was slightly lower than some PCR-based PMA viability assays (17), PD-cDDLAMP enables a substantially faster workflow with only 30-min antibiotic exposure, compared to 4 h in conventional LAMP-based AST systems (14). Notably, the method demonstrated strong performance in tetracycline resistance detection, exceeding reported accuracies of single-cell phenotyping methods for protein synthesis-targeting antibiotics (48, 60).

This study represents an initial proof-of-concept evaluation of the PMAxx-assisted PD-cDDLAMP assay for rapid antimicrobial susceptibility testing, given the relatively small number of clinical isolates evaluated. Also, β-lactamase production likely contributes to the observed resistance phenotypes.

In addition, like other NA-based phenotypic AST methods, the current PD-cDDLAMP workflow still depends on prior bacterial isolation and culture. Although the assay substantially shortens the antimicrobial susceptibility testing step, it does not eliminate the time required to obtain a pure bacterial isolate from clinical specimens, which typically requires 18–72 h (61). Furthermore, similar to other rapid molecular phenotypic AST platforms, the current workflow does not yet address the variability encountered in naturally infected clinical samples, including mixed bacterial populations and variable bacterial loads (62–64).

Future studies should therefore focus on validating the PD-cDDLAMP assay using a larger collection of clinical isolates with well-characterized and diverse resistance mechanisms. Such studies will be valuable for further validating the robustness and applicability of the assay across different resistance phenotypes and determining the influence of different resistance mechanisms on PMAxx uptake, as well as evaluating its performance in naturally infected clinical specimens. In addition, integrating upstream bacterial concentration and sample preparation approaches could facilitate direct analysis of clinical specimens (49).

Overall, PD-cDDLAMP provides a conceptual advancement in NA-based phenotypic AST by coupling PMAxx-mediated viability discrimination with colorimetric dual DNAzyme-LAMP, enabling a simplified, rapid, and broadly applicable framework for AST that reduces dependence on high-complexity nucleic acid quantification while maintaining reliable discrimination of resistant and susceptible phenotypes. These findings establish PD-cDDLAMP as a promising proof-of-concept platform and provide a strong foundation for future optimization and validation toward practical veterinary and food safety applications.

### Conclusion

Our PD-cDDLAMP-based phenotypic AST represents a significant advancement in accelerating antimicrobial susceptibility testing and highlights the potential of the assay to improve workflow efficiency. Importantly, the method maintains high analytical performance while significantly shortening the detection window compared to conventional approaches. Future work will focus on validating the assay using clinical specimens and further optimizing sample processing to enhance its translational applicability.

## Data Availability

All data supporting the findings of this study are included within the manuscript and its supplemental materials.

## SUPPLEMENTAL MATERIAL

Supplemental figures and tables spectrum.01212-26-s0001.docx Fig. S1 to S3 and Tables S1 to S4

ASM does not own the copyrights to Supplemental Material that may be linked to, or accessed through, an article. The authors have granted ASM a non-exclusive, world-wide license to publish the Supplemental Material files. Please contact the corresponding author directly for reuse.
