# Supplementary material for: PMAxx dye-assisted colorimetric dual DNAzyme-LAMP (PD-cDDLAMP) for rapid phenotypic antimicrobial susceptibility testing of Escherichia coli
Source: Microbiol Spectr. 2026 Sep 2;14(10):e01212-26. doi: 10.1128/spectrum.01212-26 (PMC13689256; doi:10.1128/spectrum.01212-26)
Supplement: Supplementary file 1 [file spectrum.01212-26-s0001.docx]

**Supplementary information**

**Fig S1:- Effect of PMAxx and Enhancer Treatment on Live and Dead E. coli Strain**
The images display the colorimetric differences observed for live and dead E. coli strains under various treatment conditions, highlighting the impact of PMAxx and enhancer treatments.

**Fig S2:-** **Visual Antibiotic Susceptibility Testing of E. coli Using Enhancer with PMAxx Under TET Exposure rather than AMP**
The image demonstrates color changes that improve antibiotic sensitivity detection in E. coli strains exposed to tetracycline, and ampicillin. This approach enhances the differentiation between resistant and susceptible E. coli strains.

**Fig S3:- Antibiotic Susceptibility Testing of E. coli Using PD-cDDLAMP Assay with AMP and TET exposure.** The photograph depicts PMAxx-treated E. coli strains, where susceptible strains exhibit a “signal-off” response, and resistant strains display a “signal-on” response, indicating differential antibiotic susceptiblity.

**Table S1. Antimicrobial resistance phenotypes and β-lactam resistance genes of the bacterial isolates included in this study.**

| **Isolate** | **Phenotypic Resistance** | **βRG (β-lactam resistance genes)** |
| --- | --- | --- |
| **AG-3** | AXO, AMP, AZI, CIP, NAL, STR, CHL, STX, TET | blaCTX-M-32 |
| **AG-1** | AXO, AMP, AZI, CIP, NAL, STR, TET | blaCTX-M-32 |
| **RM-6** | AXO, AMP, FIS | blaCTX-M-32, blaTEM-1A |
| **RM-4** | AXO, AMP, FIS, STX | blaCTX-M-32, blaTEM-1A |
| **DM-2** | AXO, AMP, CIP, NAL, GEN, STR, CHL | blaCTX-M-27, blaTEM-1C |
| **DM-15** | AXO, AMP, CIP, NAL, GEN, STR, CHL, TET | blaCTX-M-27, blaTEM-1C |
| **22-4401** | AXO, AMP, STR, CHL, FIS | blaCTX-M-32, blaTEM-1A |
| **22-4183** | AXO, AMP, TET | blaCTX-M-65 |
| **CD- Notag** | – | – |
| **CD-G11** | – | – |

**Footnote:** Antimicrobial susceptibility profiles were previously determined using commercially available 96-well microtiter plates containing 14 antimicrobial agents (Sensititre™ CMV4AGNF) and reported in a previous study (30). Phenotypic resistance is shown for antimicrobials to which each isolate was classified as resistant. βRG, β-lactam resistance genes. AXO, ceftriaxone; AMP, ampicillin; AZI, azithromycin; CIP, ciprofloxacin; NAL, nalidixic acid; STR, streptomycin; CHL, chloramphenicol; STX, sulfamethoxazole-trimethoprim; TET, tetracycline; FIS, sulfisoxazole; GEN, gentamicin. The isolates CD-Notag and CD-G11 were susceptible to all tested antimicrobials and did not harbor any detected β-lactam resistance genes.

**Table S2:- qPCR Ct values demonstrated the difference between live and dead *E. coli* strains following treatment with PMAxx and Enhancer**. Note: In the ∆Ct calculation, undetermined values were assigned a Ct value of 40**.**

| **PMAxx Dye** | **Enhancer** | **Live**  **Replicates (n=4)** | | | | **Dead**  **Replicates (n=4)** | | | |
| --- | --- | --- | --- | --- | --- | --- | --- | --- | --- |
|  |  | **1** | **2** | **3** | **4** | **1** | **2** | **3** | **4** |
| **-ve** | **-ve** | 20.817 | 20.516 | 21.153 | 20.829 | 17.604 | 17.970 | 18.267 | 17.947 |
| **+ve** | **-ve** | 21.604 | 21.699 | 21.738 | 21.681 | 34.745 | 38.128 | 34.040 | 35.638 |
| **-ve** | **+ve** | 17.763 | 17.802 | 18.169 | 17.911 | 17.919 | 17.845 | 18.392 | 18.052 |
| **+ve** | **+ve** | 22.178 | 22.279 | 22.461 | 22.306 | Un | Un | Un | Un |

**Table S3:- qPCR Ct values revealed the differences between susceptible and resistant *E. coli* strains upon exposure to ampicillin and tetracycline, both with and without PMAxx treatment**. Heat-lysed dead cells and live cells were used as quality controls. Note: In the ∆Ct calculation, undetermined values were assigned a Ct value of 40**.**

| **PMAxx Dye** | **Replicates (n=4)** | **Susceptible *E. coli* strain**  **(ATCC 25922)** | | | | **Resistant *E. coli* strain**  **(22-4183)** | | | |
| --- | --- | --- | --- | --- | --- | --- | --- | --- | --- |
|  |  | **Live** | **Dead** | **AMP** | **TET, Enh** | **Live** | **Dead** | **AMP** | **TET, Enh** |
| **-ve** | 1 | 20.873 | 17.782 | 16.407 | 19.969 | 18.688 | 17.372 | 18.315 | 18.919 |
|  | 2 | 20.873 | 17.944 | 16.488 | 20.055 | 18.758 | 17.574 | 18.365 | 19.086 |
|  | 3 | 21.230 | 18.288 | 16.818 | 20.409 | 18.929 | 17.501 | 18.435 | 19.056 |
|  | 4 | 20.992 | 18.005 | 16.571 | 20.144 | 18.792 | 17.482 | 18.371 | 19.02 |
| **+ve** | 1 | 21.613 | 37.383 | 27.084 | 32.920 | 18.958 | 33.537 | 19.270 | 20.913 |
|  | 2 | 21.861 | Un | 27.032 | 33.411 | 18.904 | 35.096 | 19.298 | 20.988 |
|  | 3 | 21.948 | 34.949 | 26.528 | 31.497 | 19.066 | 35.481 | 19.505 | 20.916 |
|  | 4 | 21.807 | 37.444 | 26.882 | 32.609 | 18.976 | 34.705 | 19.358 | 20.939 |

**Table S4:- qPCR Ct values from PMAxx-treated spiked milk samples showed differences between susceptible and resistant *E. coli* strains following exposure to ampicillin and tetracycline**. Heat-lysed dead cells, live cells, and unspiked milk samples were included as quality controls. Note: In the ∆Ct calculation, undetermined values were assigned a Ct value of 40**.**

| **PMAxx Dye** | **Replicates (n=4)** | **Unspiked milk sample** | **Spiked milk sample** | | | | | | | |
| --- | --- | --- | --- | --- | --- | --- | --- | --- | --- | --- |
|  |  |  | **Susceptible *E. coli* strain**  **(ATCC 25922)** | | | | **Resistant *E. coli* strain**  **(22-4183)** | | | |
|  |  |  | **Live** | **Dead** | **AMP** | **TET, Enh** | **Live** | **Dead** | **AMP** | **TET, Enh** |
| **-ve** | 1 | Un | 18.182 | 17.782 | 16.407 | 19.969 | 17.690 | 17.372 | 18.315 | 18.919 |
|  | 2 | Un | 18.371 | 17.944 | 16.488 | 20.055 | 17.231 | 17.574 | 17.527 | 19.086 |
|  | 3 | Un | 18.695 | 18.288 | 16.818 | 20.409 | 17.969 | 17.501 | 17.475 | 19.038 |
|  | 4 | Un | 18.416 | 18.005 | 16.571 | 20.144 | 17.63 | 17.482 | 18.371 | 19.02 |
| **+ve** | 1 | Un | 18.922 | 36.052 | 29.754 | 31.928 | 18.958 | 32.977 | 19.602 | 20.853 |
|  | 2 | Un | 19.36 | Un | 29.965 | 32.080 | 18.904 | 30.185 | 18.365 | 19.161 |
|  | 3 | Un | 19.413 | Un | 29.676 | 31.982 | 19.066 | 29.731 | 18.435 | 19.056 |
|  | 4 | Un | 19.231 | 38.684 | 29.798 | 31.997 | 18.976 | 30.964 | 19.201 | 20.351 |
